# Emergence of new types of *Theileria orientalis *in Australian cattle and possible cause of theileriosis outbreaks

**DOI:** 10.1186/1756-3305-4-22

**Published:** 2011-02-21

**Authors:** Joseph Kamau, Albertus J de Vos, Matthew Playford, Bashir Salim, Peter Kinyanjui, Chihiro Sugimoto

**Affiliations:** 1 Department of Collaboration and Education, Research Center for Zoonosis Control, Graduate School of Veterinary Medicine, Hokkaido University, Japan; 2 Department of Biochemistry, School of Medicine, University of Nairobi, P.O. BOX 30197-00100, Nairobi, Kenya; 3 Tick Fever Centre, Queensland Primary, Industries and Fisheries, 280 Grindle Rd, Wacol 4076, QLD, Australia; 4 Dawbuts pty ltd, P.O. Box 1118, Camden, NSW 2570, Australia

## Abstract

*Theileria *parasites cause a benign infection of cattle in parts of Australia where they are endemic, but have, in recent years, been suspected of being responsible for a number of outbreaks of disease in cattle near the coast of New South Wales. The objective of this study was to identify and characterize the species of *Theileria *in cattle on six farms in New South Wales where disease outbreaks have occurred, and compare with *Theileria *from three disease-free farms in Queensland that is endemic for *Theileria*. Special reference was made to sub-typing of *T. orientalis *by type-specific PCR and sequencing of the small subunit (SSU) rRNA gene, and sequence analysis of the gene encoding a polymorphic merozoite/piroplasm surface protein (MPSP) that may be under immune selection. Nucleotide sequencing of SSU rRNA and MPSP genes revealed the presence of four *Theileria *genotypes: *T. orientalis *(buffeli), *T. orientalis *(ikeda), *T. orientalis *(chitose) and *T. orientalis *type 4 (MPSP) or type C (SSU rRNA). The majority of animals showed mixed infections while a few showed single infection. When MPSP nucleotide sequences were translated into amino acids, base transition did not change amino acid composition of the protein product, suggesting possible silent polymorphism. The occurrence of ikeda and type 4 (type C) previously not reported to occur and silent mutation is thought to have enhanced parasite evasion of the host immune response causing the outbreak.

## Background

*Theileria *was first recorded in cattle in Australia in 1910 [1] and infection rates in endemic parts of the eastern states (QLD, NSW, Victoria) are high. In a survey in one region of New South Wales (NSW), 60% of cattle were positive on blood smears [1] while cattle in endemic parts of Queensland (QLD) showed herd and animal seroprevalences of 75% and 41% respectively [2]. Infection is usually non-pathogenic but it was recognized more than 40 years ago that under certain, undefined conditions, the organism may become highly virulent [1]. Isolates of *Theileria*, identified as *T. buffeli*, have been recorded as common intra-erythrocytic parasites that are spread by members of the *Haemaphysalis *tick genus, with wide distribution throughout eastern Australia [3]. Due to the fact that only a few cases of clinical disease associated with *Theileria *infection had been reported prior to 2005, and transmission studies failed to elicit clinical disease in test subjects, Australian strains of *Theileria *were considered to be benign [3-5]. In recent years, a number of outbreaks were reported in cattle near the coast of NSW, characterized by anemia, jaundice, depression, abortion, mortality and the presence of *Theileria *in blood films [6]. Most of the outbreaks were seen in periparturient cattle that have recently been moved from inland to coastal areas.

The *Theileria *species present in Australia is referred to as *T. buffeli *[7] and belongs to the *T. orientalis/sergenti/buffeli *group of generally benign, cosmopolitan parasites found in many countries [8-12]. The taxonomic status of this group has been debated for many years. Based on serological and morphological grounds and transmission experiments, [3] suggested these parasites all belong to one species, *T. orientalis*, despite the fact that the *Theileria *sp. present in Japan, Korea and Russia is locally known as *T. sergenti. T. sergenti *is an invalid name taxonomically since it has been used to previously describe a parasite of sheep [13,14]. Based on Major Piroplasm Surface Protein (MPSP) and 18S rDNA sequences, studies have designated these parasites as the *T. sergenti/T. buffeli/T. orientalis *group of benign theileria [15,16]. Comparison of the MPSP sequences of these parasites have shown that global parasites of this group can be classified into 1-8 types [17,18]. However, based on the MPSP sequence, one benign Theileria parasite from Brisbane, Australia (*T. buffeli*, Warwick) may as yet be in an unclassified group [18]. Within the pathogenic protozoans the most diverging proteins are those at the parasite surface that mediate physical recognition, response and modulation of the host [19]. In this regard, there was a need to amplify and sequence the MPSP gene to assess the antigenic variation in order shed light on how parasite is evolving in evading the host system.

At the onset of the outbreak, preliminary investigations confirmed the presence of parasite surface antigens identical to those found in outbreaks of clinical theileriosis in Japan. These antigens had not been previously found in Australian *Theileria *isolates [6]. The purpose of this study was to confirm if the antigens detected reflected the presence of parasites similar to those found in Japan, determine if the outbreaks reported in NSW was associated with an identifiable pathogen; and finally determine whether the *Theileria *species identified in the affected cattle are phylogenetically related to any of the 8 types such as *T. buffeli *(Warwick), to chitose type or, more notably, to ikeda type which is commonly found in Japan and Korea from anemic cattle suffering from bovine piroplasmosis. For the sake of simplicity, we will refer to the parasites as type 1-8 when using MPSP sequences.

## Materials and methods

### Samples collection and DNA extraction

**Figure 1:**
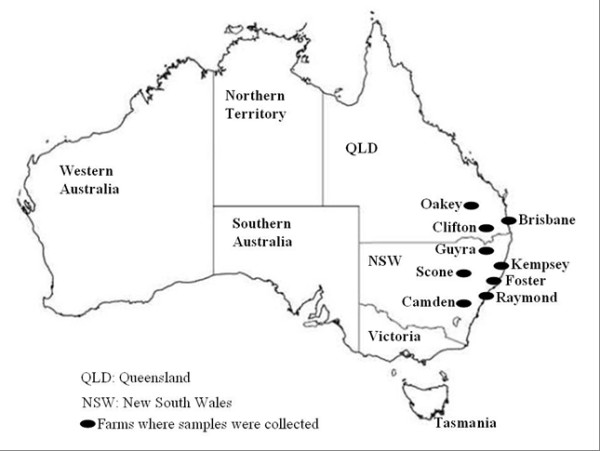
**Australian map showing sampling locations in two states of Queensland and New South Wales**. There were nine farms in total, three in Queensland and six in New South Wales. All the farms were in the coastal eastern part of Australia as indicated in the map.

Blood samples were collected from cattle of mixed ages known or suspected to be infected with *Theileria *in six farms in NSW and three farms in QLD. Sample locations are as indicated in the map Figure 1. Of the 45 animals sampled, 17 were from QLD and 28 from NSW. Parasite DNA was extracted from whole blood samples using Qiagen QIAamp DNA Blood Kits (Qiagen, USA) and stored at -20°C until use.

### Investigation of alternative causes of haemolytic anaemia

To investigate causes of haemolytic anaemia other than theileriosis, other alternatives considered and eliminated included babesiosis, anaplasmosis and trypanosomiasis. All cases were tested for the causative agents by smear examination and turned out to be negative. Chronic copper poisoning was eliminated because copper levels of affected cattle were in the normal range and none of the changes consistent with copper poisoning were seen on histopathology in the liver or kidney of autopsied cattle [6]. Other ingested/injected substances from grazing were excluded because there was no history of access/injection to such pastures. Immune mediated Autoimmune haemolytic anaemia in calves less than 6 months of age was discounted because most cases were diagnosed in cattle >6 months of age. Other causes of haemoglobinuria e.g. Bacillary haemoglobinuria (caused by *Clostridium haemolyticum*), Postparturient haemoglobinuria (a manifestation of low dietary phosphate intake) and leptospirosis were all eliminated as haemoglobinuria was not seen in any cases and tests for leptospirosis were negative or low consistent with vaccination. Bovine virus disease (BVD) is occasionally associated with anaemia but tested animals were negative.

### Polymerase chain reaction and sequencing

The genomic DNA was subjected to PCR and sequencing for identification of the *Theileria *species and types present. The PCR primers used in this study are given in Table 1. Two genes were targeted for PCR: SSU rRNA and MPSP. For the ssu rRNA gene, three type-specific sets of primers were used, each specific for one of the ikeda, chitose and buffeli types of the *T. sergenti/T. buffeli/T. orientalis *group [20,21]. In addition universal *Theileria *SSU rRNA gene primers were used to PCR amplify the full-length gene of approximately 1800 bp, which was then cloned into a plasmid vector for sequencing. Internal ssu rRNA gene primers were used to facilitate sequencing of the cloned fragment [22]. In case of MPSP gene, a 776 bp fragment PCR amplified using primers universal for *T. sergenti/T. buffeli/T. orientalis *group. Conditions used for PCR amplifications were 10 μl of GoTaq Master Mix (Promega, Madison, WI, USA); 10 mM of each primer scaled up to 20 μl reaction mix. MPSP PCR products were then cloned into plasmid and sequenced. Thirty cycles of PCR were carried out using thermocycler (Takara co. Japan) with the following conditions: denaturation at 95°C, for 15 s; annealing at 57°C for 30 s; and extension at 72°C for 1 min. The PCR products of expected sizes were confirmed by electrophoresis on 1% agarose gel, visualized under UV and then ligated into pGEMT.

**Table 1:** Primers used in identification of *Theileria orientalis *types

Primer	Sequences	Expected Size
**SSU rRNA *T. orientalis *Type specific Forward Primer**		
Ts-Ikeda	5'-AAGGATCCGTCTCTGCTACCGCCGC-3'	826**bp **(Kubota et al., 1996)
Ts-Chitose	5'-GCGGATCCTCATCGTCTCTGCAACT-3'	831**bp "**
Ts-Buffeli	5'GCGGATCCGCTCTGCAACCGCAGAG-3'	825**bp "**
**Type specific Universal Reverse Primer**		
Ts-R	5'-TGTGAGACTCAATGCGCCTA-3'	
**MPSP **( Targeting entire ***Theileria orientalis *group**)		
MPSP Forward Primer	5'-CTTTGCCTAGGATACTTCCT-3'	776**bp (**Ota et al., 2009)
MPSP Reverse Primer	5'-ACGGCAAGTGGTGAGAACT-3'	"
**SSU **(***Theileria orientalis***)		
SSU rRNA Forward Primer	5'-ACCTGGTTGATCCTGCCAGT-3'	1800**bp **(Chae et al., 1998)
SSU rRNA Reverse Primer	5'-TAGGAAGACGTCCAAGTGGAATG-3'	"
SSU rRNA **Internal **Primers (F&R)	F-5' ATTGGAGGGCAAGTCTGGTG-3'	700**bp **(This study)
	R-5'- CTCTCGGCCAAGGATAAACTCG-3'	"

### SSU rRNA Gene sequence analysis

The samples selected for SSU rRNA gene sequencing analysis were from herds with high parasitemia (1-7%) and or those herds with reported cases of deaths. To minimize the amplification errors caused by *Taq *polymerase and increase the accuracy, a minimum of 5 clones from two PCR products were subjected to nucleotide sequencing. The complete nucleotide sequences were determined from both strands using 3130 XL Genetic Analyzer or 3130 Genetic Analyzer with the BigDyes Terminator cycle sequencing kit (Applied Biosystems). Nucleotide sequences were applied to a Basic Local Alignment Search Tool (BLAST) in NCBI for homology analyses of *Theileria *MPSP genes. The sequences were edited using Genetyx Ver. 9 (http://www.sdc.co.jp/genetyx/) and aligned by clustalX2. The Neighbor Joining (NJ) phylogenetic tree was constructed using MEGA4 [23]. The molecular distances were estimated by the Kimura two parameter models [24]. The nodes were tested for robustness by 1000 bootstrap replications [25]. *T. annulata *and *T. parva *served as the out group. The representative sequences obtained were registered in the DDBJ/EMBL/Genbank nucleotide sequence database under the assigned accession numbers AB520953-AB520958.

### MPSP Gene sequence analysis

Further analysis to examine the diversity of the MPSP gene in samples representing the localities in NSW and QLD was undertaken. Based on diagnostic PCR and ssu rRNA sequences, samples for MPSP gene sequence analysis were selected considering their geographic origin and *T. orientalis *type detected by ssu rRNA sequence analysis. A total of 20 samples were sequenced using MPSP gene analysis. DNA sequencing analysis of positive clones was carried out to examine the diversity of the MPSP gene in these samples. Universal primers [6] capable to detecting all eight different types of *T. orientalis *types for MPSP allele amplifying 776-bp *T. orientalis *were used in sequencing.

## Results

### Diagnostic PCR using SSU rRNA species-specific primers

#### New South Wales

The diagnostic PCR detected ikeda and chitose types In Kempsey property. On this property, there were eight positive cases, five with single ikeda infection, one case with chitose type while the others were mixed infection of ikeda and chitose. Parasitemia ranged from 1-7% with one case of fatality reported. In Guyra properties, there were six positive animals in total. Two animals had either buffeli or chitose type while sixth one had co-infection of buffeli and chitose types. In Foster locations, there were three positive cases identified, two of which had mixed infection of chitose and ikeda, while the third one had mixed infections of all the three *theileria orientalis *types. Seven cases of fatalities were recorded in this property. The Scone property was negative for ikeda, but three cases were positive with chitose single infection while the other had mixed infection of chitose and buffeli. The Camden property had two positive cases with ikeda. On this property parasitemia ranged 2-7% and no fatality was reported. Percentage of *T. orientalis *types detected in New South Wales was ikeda (46%), chitose (46%) and 17.9% buffeli, majority of which mixed infections of ikeda and chitose.

#### Queensland

There were twelve animals sampled from a Brisbane property. Of these two had single infection with buffeli, three were positive with ikeda and chitose mixed infection, while four had mixed infection of chitose and buffeli types. Three animals were negative. Clifton property had three positive animals with mixed infection of chitose and buffeli types. There was only one sample from Oakey collected which was identified positive with buffeli type of infection. Percentage of *T. orientalis *types detected in Queensland was ikeda (5.9%), chitose (52.9%) and 58.8% buffeli, majority of which mixed infections of chitose and buffeli. The summary of the diagnostic PCR result is described in Tables 2 and 3.

**Table 2:** List of isolate samples from outbreak regions in New South Wales, Australia, including geographic origin, *Theileria orientalis *type(s) identified by specific PCR and herd clinical history

**Sample No**.	Location	Ikeda	Chitose	Buffeli	Herd Clinical History
1*♦	Raymond Terrace	-	**+**	**+**	Anemia, Jaundice

2*	Forster location 1	**+**	**+**	**+**	Shorthorn, 1 dead of 40, 1 sick

3*	Forster location 2	**+**	**+**	-	Angus, 6 of 60 dead, 8 sick, anemia and jaundice

4* ♦	Forster location 2	**+**	**+**	-	"

5*	Kempsey	**+**	**+**	-	Friesian, 1 dead of 150, 3 sick. Moved to property 2 months earlier. Anemia, jaundice, condition loss. Parasitemia 1-7%

6*♦	Kempsey	-	**+**	-	"

7*	Kempsey	**+**	-	-	"

8*	Kempsey	**+**	-	-	"

9	Kempsey	**+**	-	-	"

10*♦	Kempsey	**+**	-	-	"

11	Kempsey	**+**	-	-	"

12*	Kempsey	-	**+**	-	"

13	Guyra location 1	-	-	**+**	Cattle moved to property recently, some sick. Parasitemia of sampled cattle unknown

14	Guyra location 1	-	-	-	"

15*	Guyra location 1	**+**	-	-	"

16	Guyra location 1	-	-	-	"

17*	Guyra location 1	**+**	-	-	"

18	Guyra location 1	-	-	-	"

19	Guyra location 2	-	-	-	Hereford, 3 adults of 250 dead, 2 sick. Moved to property 3 months earlier. Anemia, abortion. Parasitemia of sampled animals unknown.

20	Guyra location 2	-	**+**	**+**	"

21*	Guyra location 2	-	**+**	-	"

22	Scone	-	**+**	**+**	Angus, 8 dead of 180 cows, 12 sick. Moved to property 10 months earlier. Anemia, jaundice. Parasitemia 1-7%

23	Scone	-	**+**	-	"

24*	Scone	-	**+**	-	"
25*	Scone	-	**+**	-	"

26*	Camden	**+**	-	-	Friesians, adults with jaundice, Anemia and fever after calving. Parasitemia 2-7%

27	Camden	-	-	-	"

28*♦	Camden	**+**	-	-	

**Total Percentages**		**46.4%**	**46.4%**	**17.9%**	

*Samples sequenced with MPSP gene

♦Samples sequenced with SSU rRNA gene

**Table 3:** List of isolate samples from Non-outbreak regions in Queensland, Australia, including geographic origin, *Theileria orientalis *type(s) identified by specific PCR, and herd clinical history

**Sample No**.	Location	Ikeda	Chitose	Buffeli	Clinical History
29	Brisbane	-	-	-	N/A

30	Brisbane	-	-	-	N/A

31	Brisbane	-	-	-	

32	Brisbane	**+**	**+**	-	Heavy infection only after splenectomy

33	Brisbane	**+**	-	-	"

34	Brisbane	-	**+**	**+**	"

35*	Brisbane	**+**	**+**	-	"

36	Brisbane	-	**+**	**+**	"

37	Brisbane	-	**+**	**+**	"

38	Brisbane	-	-	**+**	"

39	Brisbane	-	-	**+**	"

40	Brisbane	-	**+**	**+**	"

41	Clifton	-	-	-	N/A

42	Clifton	-	**+**	**+**	Cattle originating from Clifton and Oakey, SE Queensland stationed at Tick Fever Center

43	Clifton	-	**+**	**+**	"

44*♦	Clifton	-	**+**	**+**	"

45*	Oakey	-	-	**+**	"

**Total Percentages**		**5.9%**	**52.9%**	**58.8%**	

*Samples sequenced with MPSP gene

♦Samples sequenced with SSU rRNA gene

## Small subunit ribosomal RNA (SSU rRNA) sequence analysis

**Figure 2:**
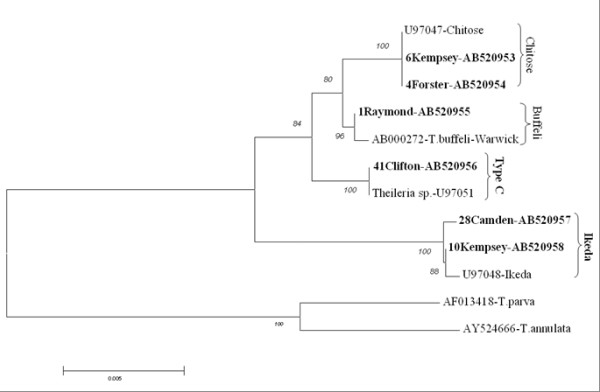
**Phylogenetic relationships among *Theileria *types isolated in NSW and QLD based on SSU rRNA sequences**. The tree was constructed using the neighbor-joining algorithm with molecular distances estimated by the Kimura-2 parameter model. This tree shows chitose, buffeli and ikeda types in 3 different clades. One corresponding sequence each from *T. parva *(AF013418) and *T. annulata *(AY524666) served as outgroups. Bootstrap values are shown as percentages at each node based on 1000 replicates. Branch lengths correlate to the number of substitutions inferred according to the scale shown.

The sequence results (Figure 2) revealed the presence of four types of *Theileria*, type 2 (ikeda), type 1 (chitose), type 3 (buffeli) and type C. Previously type C has been reported to occur in Japan and Korea [26]. When BLAST was done, animal numbers 4 (Forster) and 6 (Kempsey) showed high identity to *Theileria *chitose-type. Number 1 animal from Raymond Terrace showed high identity to *Theileria *buffeli-type of accession AB000272. Animal 41-Clifton was closely related to type C accession number U97051. Animal number 10 from Kempsey and 28 from Camden showed high identity to *Theileria *ikeda-type of accession U97048. Based on SSU rRNA sequences, the three types [26,27] were confirmed in addition indentifying type C.

## Major piroplasm surface protein (MPSP) sequence analysis

The MPSP phylogenetic tree (Figure 2) was drawn based on nucleotides sequences. There was a number of single nucleotide polymorphism (SNPs) observed within and between group types. For example, (type 1) the chitose group had three clades within the group while type 2 (ikeda) had four clades forming the larger type 2 (ikeda). All these different clades within the group were as result of SNPs occurring. When the nucleotides were translated in protein, they did not result into amino acid profile change. Of interest to note are the sequences from animals number 41-Clifton and 45-Oakey which showed mosaic kind of pattern forming repeated sequences of chitose and ikeda suggesting the possibility of genetic recombination occurring. When BLAST was done, sample number 5, 6 and 12 from (Kempsey), 24 and 25 (Scone), 3 and 4 (Forster), 21 (Guyra) and 35 (Brisbane) all showed high identity to the registered database sequences of *Theileria *chitose-type accession D12689. Sample number 41 and 45, Clifton and Oakey respectively clustered with the classical benign type 4 D87201-Cheju-Korea, this type 4 is commonly found in Cheju Island in Korea but recent has been reported to occur in Okinawa island of Japan [6]. Samples 2 (Forster), 7 and 8 (Kempsey) and 27 (Guyra) showed high identity to ikeda types (AB218433 and D11046). In addition to high similarities revealed within each group of the four *T. orientalis *types, there were polymorphism differences observed within a specific type group. For example, in type 1 (chitose), three distantly groups were found but falling within the larger type 1 group. Single nucleotide polymorphism (SNPs) within subsections in a group led to these sub-groups. The type 2-ikeda group had four sub-groups with animal number 15 Guyra being distantly from the rest of the larger ikeda group. The sample 1Raymond, although grouped in type 3-buffeli was distantly related to typical buffeli as shown by the long bar.

## Discussion

Historically *Theileria *infection has not been associated with significant disease in Australian cattle. Consequently the identification of *Theileria *in blood smears from diseased cattle was initially dismissed as being unrelated to the disease outbreaks and investigations concentrated on identifying other possible causes. It was only when all other possible causes had been eliminated that a diagnosis of theileriosis was made. Consequently preliminary investigations had detected presence of parasite surface antigens identical to those found in clinical theileriosis in Japan. These parasite surface antigens were confirmed by the detection and subsequent identification of the ikeda, chitose and type 4/type C isolates commonly associated with clinical cases in Japan and Korea. This may explain partly the cause of theileriosis.

There have been several epidemiological studies on theileriosis (*T. orientalis*) in many countries such as Turkey, France, Spain [28-31], but only a few fatal cases due to *T. buffeli *have been reported to date [8,32,33]. Of the few cases reported, one fatal case was suspected that the clinical theileriosis might have been exacerbated by other factors compromising the immune competence of the animal such as concurrent bovine leukemia virus infection. In the study all possible causes of regenerative anemia such as babesiosis, anaplasmosis and trypanosomosis, bovine virus disease among others were eliminated.

*T. orientalis *of which ikeda type is a representative, seems to be closely associated with clinical cases in Japan and other Asian countries [34,35]. In New South Wales the epicenter of theileriosis outbreak, ikeda and chitose types were the predominant types detected accounting for 46.4% each. In contrast, Queensland had only 5.9% ikeda with and buffeli at 58.8%. No case of theileriosis outbreak reported in Queensland. From our diagnostic PCR, the results indicate presence of ikeda may have been cause of theileriosis outbreak resulting in severe anaemia, high parasitemia and cases of deaths reported in some properties.

**Figure 3:**
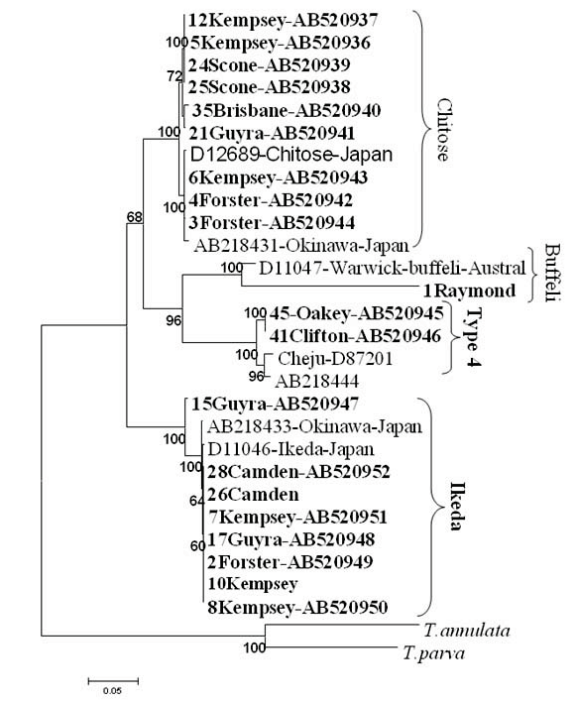
**Phylogenetic relationships among *Theileria *isolated in NSW and QLD based on MPSP sequences**. The tree was constructed using the neighbor-joining algorithm with molecular distances estimated by the Kimura-2 parameter model; three clades are noted representing chitose, buffeli and ikeda types. *T. parva *and *T. annulata *served as outgroup. Bootstrap values are shown as percentages at each node based on 1000 replicates. Branch lengths correlate to the number of substitutions inferred according to the scale shown.

Clear taxonomical discrimination of parasites of this group, often referred as "*T. orientalis/buffeli*" has not been well established. As shown in Figure 3, parasites found in Australia in this study clearly separated into four clades (types) that are represented by type 2 (ikeda), type1 (chitose), type 3 (buffeli) and type 4 based on MPSP sequences. Based on MPSP gene, animal 15 Guyra (AB520947) sequence is marginally separated from ikeda and other Australian parasites in this clade indicating increased number of SNPs observed when compared to other members in this group. There was evidence of SNPs observed in all the four type groups, though base transition did not result in amino acid change, suggesting silent mutation happening on the sequences coding for MPSP gene [36]. Provide evidence that silent SNPs can affect *in vivo *protein folding and, consequently, function that may in this case cause the variation of type of antigen detected. Silent mutation may explain the unique parasite surface antigens detected earlier in the preliminary investigations [6]. We hypothesize that this unique antigenic variation might have enhanced parasite's ability to evade host immune system [37] causing the parasite to become more virulent.

Many of the samples examined showed mixed populations of the different *Theileria *types. This is a common phenomenon that has also been noted in Japan [20,38], Korea [39] and Thailand [40] but its clinical significance is unknown. Mixed infections can occur both in the host or transmitting vector which results in different types interacting resulting in mutation due to competition to propagate and or evade host system. Recombination has been shown to occur frequently in *Theileria *species and has been described to occur within Tams 1 gene of *T. annulata*, homologue to MPSP in *T. orientalis *[41,42]. The possibility of genetic exchange where recombination result in an almost shuffling of sequence types is common. Our close examination of the MPSP sequence types when linked to SSU rRNA provided evidence of genetic re-assortment i.e. when the MPSP sequences were linked to the same SSU rRNA sequence. The mode of vector transmission also might have played a part in the genetic re-assortment as observed in the MPSP gene sequence analysis [43]. Suggested that mixed infections are thought to disturb the host's immune system, since MPSP of ikeda and chitose are not serologically cross-reactive.

Unlike the sporadic cases seen in the past, the recent outbreaks featured multiple cases, severe symptoms including aborted foetuses and many deaths in cattle of all ages with some individual herds losing 30% of affected mobs. The majority of outbreaks in coastal areas have been in cattle that had been introduced from tick-free areas 4 to 6 weeks previously. The non-coastal outbreaks have mostly involved home-bred cattle on properties where coastal cattle had been introduced a few months previously. Global warming has been implicated in emergence of pathogens [44] that are adaptable or transmissible by vectors that otherwise would not have been possible. Changes in farm management including animal movements that accompany prolonged drought may also have compounded the outbreak of theileriosis.

## Authors' contributions

JK performed the experiments, data analysis and manuscript preparation. AJ conceived of the study, participated in the design and coordination of the experiment, provided with DNA samples from Queensland and helped draft of the manuscript. MP participated in the design and coordination of the experiment, provided with DNA samples from New South Wales, investigated alternative causes of haemolytic anaemia and helped draft manuscript. BS helped with construction of phylogenetic trees and interpretation. PK helped in revision of the manuscript. CS participated in the design of the experiment, supervisory role of the molecular work, provided the consumables and reagents required and helped in review of the manuscript. All authors read and approved the final manuscript.

## Competing interests

The authors declare that they have no competing interests.
